# Genome-scale identification of Soybean BURP domain-containing genes and their expression under stress treatments

**DOI:** 10.1186/1471-2229-10-197

**Published:** 2010-09-13

**Authors:** Hongliang Xu, Yaxuan Li, Yueming Yan, Ke Wang, Ya Gao, Yingkao Hu

**Affiliations:** 1 College of Life Sciences, Capital Normal University, Beijing, 100048, China

## Abstract

**Background:**

Multiple proteins containing BURP domain have been identified in many different plant species, but not in any other organisms. To date, the molecular function of the BURP domain is still unknown, and no systematic analysis and expression profiling of the gene family in soybean (*Glycine max*) has been reported.

**Results:**

In this study, multiple bioinformatics approaches were employed to identify all the members of BURP family genes in soybean. A total of 23 BURP gene types were identified. These genes had diverse structures and were distributed on chromosome 1, 2, 4, 6, 7, 8, 11, 12, 13, 14, and 18. Phylogenetic analysis suggested that these BURP family genes could be classified into 5 subfamilies, and one of which defines a new subfamily, BURPV. Quantitative real-time PCR (qRT-PCR) analysis of transcript levels showed that 15 of the 23 genes had no expression specificity; 7 of them were specifically expressed in some of the tissues; and one of them was not expressed in any of the tissues or organs studied. The results of stress treatments showed that 17 of the 23 identified BURP family genes responded to at least one of the three stress treatments; 6 of them were not influenced by stress treatments even though a stress related *cis*-element was identified in the promoter region. No stress related *cis*-elements were found in promoter region of any BURPV member. However, qRT-PCR results indicated that all members from BURPV responded to at least one of the three stress treatments. More significantly, the members from the RD22-like subfamily showed no tissue-specific expression and they all responded to each of the three stress treatments.

**Conclusions:**

We have identified and classified all the BURP domain-containing genes in soybean. Their expression patterns in different tissues and under different stress treatments were detected using qRT-PCR. 15 out of 23 BURP genes in soybean had no tissue-specific expression, while 17 out of them were stress-responsive. The data provided an insight into the evolution of the gene family and suggested that many BURP family genes may be important for plants responding to stress conditions.

## Background

The BURP domain-containing protein family is defined by its conserved amino acid motif whose name is based on four typical members, BNM2, USP, RD22, and PG1β. BURP domain-containing proteins have so far only been found in plants, suggesting that their functions may be plant specific. Generally, the BURP family proteins consist of several modules: an N-terminal hydrophobic domain, a presumptive transit peptide; a variable internal region containing either a short conserved segment or other segments; an optional segment consisting of repeated units which is unique to each member; and the BURP domain at the C-terminus [1].

BURP domain-containing proteins were classified into four subfamilies, BNM2-like, USP-like, RD22-like, and PG1β-like [2]. All members of each subfamily contain BURP domain at the C-terminal region. Within the domain there are several conserved amino acid residues, including four cystein-histidine repeats and one tryptophan residue. The spacing between the four CH residues is highly conserved, being X_5_-CH-X_10_-CH-X_23-27_-CH-X_23-26_-CH-X_8_-W, where X is any amino acid. The difference between the BURP domain-containing proteins mostly occurred in the region immediately downstream of the hydrophobic signal peptide. This region contains a short conserved segment and an optional segment of repeated units. Unlike other BURP domain-containing proteins, the BNM2-like subfamily proteins are directly linked to the C-terminal region by a short conserved segment following the signal peptide [3]. Both the USP-like subfamily and RD22-like subfamily proteins can be distinguished from other subfamily proteins by a region containing ~30 amino acid residues followed by a variable region. However, for the RD22-like subfamily proteins, the variable region consists of repeat sequences while USP-like subfamily has no such a region [2]. The PG1β-like family proteins differ from other subfamily members by the presence of multiple copies of a 14 amino-acid repeat sequence [4].

Many members of the BURP protein family have been found in various plant species but the functions of these proteins are unknown or only tentatively explored. Transcription of *BNM2 *from oilseed rape (*Brassica napus L*.) is induced at the start of microspore embryogenesis but the corresponding protein remains confined to seeds where it is localized in the protein storage vacuoles [4-7]. VfUSP, an abundant non-storage seed protein with unknown function from the field bean (*Vicia faba L*.), is expressed during the early stages of zygotic embryogenesis [8], and at very early stages of in vitro embryogenesis [9]. *ASG1 *is a specifically expressed gene during the early embryo sac development in apomictic gynoecia, but does not express in sexual gynoecia of Panicum [10]. PG1β, the non-catalytic β-subunit of the polygalacturonase isozyme (PG) from ripening tomato (*Solanum lycopersicum *L.) plays a significant role in regulating pectin metabolism by limiting the extent of pectin solubilization and depolymerization [4,11]. SCB1 is a seed coat specific protein [12] identified in soybean. OsRAFTIN1, an anther-specific protein in rice (*Oryza sativa L*.), transports sporopollenin from tapetum to developing microspores via Ubisch bodies [13]. AtUSPL1 occurs in cellular compartments such as Golgi cisternae, dense vesicles, prevaculoar vesicles and the protein storage vacuoles in the parenchyma cell of cotyledons, and thus may play a role in seed development [14]. So it seems that many BURP family members play a role in maintaining the normal metabolism or development in plants.

Besides their significant roles in plant development and metabolism, many BURP domain-containing proteins have been reported to be stress-induced. *RD22*, a drought induced protein of *Arabidopsis *[15], has often been used as a reference for drought stress treatment in different plants. The mechanism for abscisic acid (ABA) regulation of plants stress has been well studied in *Arabidopsis*. ABA activates the gene expression of *rd22BP1 *and *ATMYB2*, which in return induces the expression of the *RD22 *gene as transcription factors [16]. Both ADR6, the auxin down-regulated protein [17], and SALI3-2, an aluminium-induced protein [18], were found in soybean. Transcription of *BnBDC1*, from oilseed rape, was up-regulated by manitol, NaCl and ABA, and down-regulated by UV irradiation and salicylic acid [19]. Among the 17 BURP genes from *Oryza sativa*, 15 were induced by at least one of the stresses including drought, salt, cold, and ABA treatment [3]. All the reports about BURP family genes indicate that this group of genes may have two major functions in plants. One is the regulation of reproductive development in plants, such as *BNM2*, *VfUSP *and *ASG1*, while the other is responsive to stress, for example RD22 and ADR6.

Soybean is one of the most economically and nutritionally important crops. It provides not only vegetable protein and edible oil but also essential amino acids for humans and animals. However, soybean production is threatened by drought and other environmental stresses. BURP domain-containing proteins are known to be involved in embryogenesis or stress responses. Over the years, some of the soybean BURP proteins including ADR6, SALI3-2 and SCB1 have been identified and studied. As the genome sequence of the soybean is complete, it is possible to analyze the entire family of soybean BURP proteins. In the current study, 23 putative genes of the BURP family were identified. To discover the functions of all the members, we investigated the transcript level of all genes in eight different tissues and organs as well as under 3 different stress treatments. The results presented in this study showed that the expression of most of the soybean BURP genes is non-tissue-specific but stress-responsive.

## Results

### Identification and distribution of soybean BURP family members

**Figure 1:**
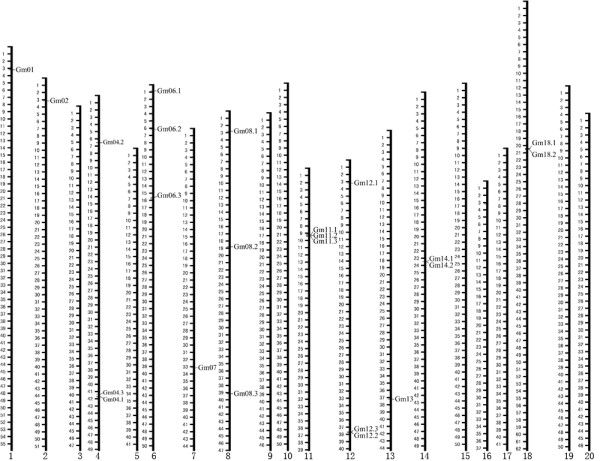
**Genomic distribution of GmBURP genes on *Glycine max *chromosomes**. The bars represent the loci with gene name on the right. (Scale in Mbp).

**Figure 2:**
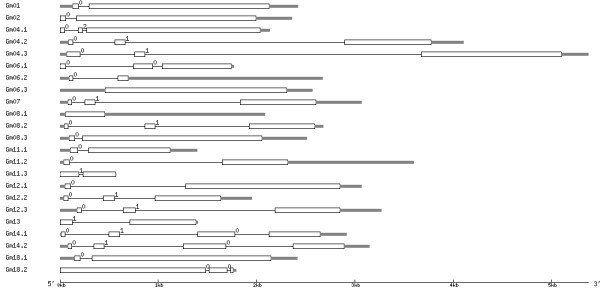
**The intron-exon structures of *GmBURP *genes**. The white boxes, exons; fine lines, introns; thick grey lines, UTR (Un-translated regions). Names for the genes are on the left.

Through soybean genome blast and online software identification, a total of 23 putative BURP genes were identified. These putative genes were designated *Gm01*, *Gm02*, *Gm04.1*, *Gm04.2*, *Gm04.3*, *Gm06.1*, *Gm06.2*, *Gm06.3*, *Gm07*, *Gm08.1*, *Gm08.2*, *Gm08.3*, *Gm11.1*, *Gm11.2*, *Gm12.3*, *Gm11.3*, *Gm12.1*, *Gm12.2*, *Gm13*, *Gm14.1*, *Gm14.2*, *Gm18.1*, *Gm18.2*. Previously reported BURP domain-containing protein genes in soybean, *SCB1*, *ADR6*, *BURP2*, and *Sali3-2 *correspond to *Gm07*, *Gm12.2*, *Gm14.1*, and *Gm12.3*, respectively. The 23 soybean BURP genes were located on chromosome 1, 2, 4, 6, 7, 8, 11, 12, 13, 14, and 18 (Figure 1). We noticed that BURP genes on chromosome 11 and 18 clustered together with each other. Examination of the location of each BURP gene http://soybase.org/gbrowse/cgi-bin/gbrowse/gmax1.01/ revealed that all *GmBURP *genes except for *Gm07*, *Gm08*.*2*, *Gm14*.*1*, and *Gm14*.*2*, were located in duplicate regions (Table 1). *Gm04*.*3*, *Gm06*.*1*, *Gm08.1*, *Gm11*.*1*, and *Gm18*.*1 *were found as single copy in their duplicated areas. The results of intron-exon structure identification (Figure 2) showed that most of BURP domain-containing protein genes have very few introns, 10 out of 23 have only one intron; 9 have two introns; and 2 have no introns. The other two genes have 3 introns. BURP genes located close to each other on the same chromosome tend to have similar structures. Specifically, both *Gm4.1 *and *Gm4.3 *have one intron flanked by two exons; *Gm11.1*, *Gm11.2*, and *Gm11.3 *have one intron; *Gm14.1 *and *Gm14.2 *have 3 introns. However, *Gm18.1 *has one intron and the nearby *Gm18*.*2 *has two introns.

**Table 1:** Summary of *GmBURP *family members

Gene Name	Protein Name	Locus	Intron bp	Signal peptide AA	Protein			Duplicate gene
					AA	MM	PI	
BNM2								
*Gm06.1*	Gm06.1	Glyma06g12570.1	786	None	319	35976.3	6.61	*
*Gm11.1*	Gm11.1	Glyma11g12670.1	476	33	300	33882.98	7.00	*
*Gm11.2*	Gm11.2	Glyma11g12770.1	1940	24	538	59980.87	6.26	*Gm12.1*
*Gm11.3*	Gm11.3	Glyma11g12780.1	43	None	174	19700.08	8.89	*Gm12.1*
*Gm12.1*	Gm12.1	Glyma12g04880.1	1404	24	542	59692.35	5.98	*Gm11.3*
USP								
*Gm08.1*	Gm08.1	Glyma08g04080.1	297	None	133	14618.93	7.73	*
*Gm08.2*	Gm08.2	Glyma08g24780.1	1828	21	271	30265.01	8.07	**
*Gm12.2*	Gm12.2	Glyma12g34500.1	1103	19	273	30422.98	6.36	*Gm13*
*Gm12.3*	Gm12.3	Glyma12g34570.1	2394	19	277	31881.42	5.80	*Gm13*
*Gm13*	Gm13	Glyma13g35970.1	589	None	264	29416.73	6.89	*Gm12.2*
RD22								
*Gm04.2*	Gm04.2	Glyma04g08410.1	3025	21	342	36992.01	6.94	*Gm06.2*
*Gm06.2*	Gm06.2	Glyma06g08540.1	2969	21	344	37209.5	7.69	*Gm04.2*
*Gm14.1*	Gm14.1	Glyma14g20440.1	1830	22	350	37982.92	6.05	**
*Gm14.2*	Gm14.2	Glyma14g20450.1	2006	21	368	39617.04	8.54	**
*Gm18.1*	Gm18.1	Glyma18g18980.1	517	22	623	67867.96	6.21	*
PG1Beta								
*Gm01*	Gm01	Glyma01g03760.1	499	24	630	68200.03	8.75	*Gm02*
*Gm02*	Gm02	Glyma02g03960.1	443	25	629	68307.25	8.31	*Gm01*
*Gm04.1*	Gm04.1	Glyma04g35360.1	252	20	618	67759.47	9.08	*Gm06.3*
*Gm06.3*	Gm06.3	Glyma06g19480.1	690	None	614	67293.46	8.95	*Gm04.1*
*Gm08.3*	Gm08.3	Glyma08g39700.1	597	23	628	68322.61	8.38	*Gm18.2*
*Gm18.2*	Gm18.2	Glyma18g19040.1	68	None	565	61967.33	8.24	*Gm08.3*
BURPV								
*Gm04.3*	Gm04.3	Glyma04g35130.1	3642	None	554	62422.49	7.23	*
*Gm07*	Gm07	Glyma07g28940.1	2114	21	306	34164.28	7.64	**

In *Glycine max*, the genes encoding BURP domain-containing proteins were named according to their loci prefixed by "Gm"

** Genes located outside of the duplicated regions

* Genes located in duplicated regions but were found only in single copy

### Sequence analysis of BURP proteins

The BURP domain for each predicted protein was identified by searching against the SMART database. One of 23 BURP domain-containing proteins, Gm08.1 had only an incomplete BURP domain at the C-terminal. The characteristics of the soybean BURP proteins including the signal peptide, pI, molecular weight, and some additional gene features are presented in Table 1.

**Figure 3:**
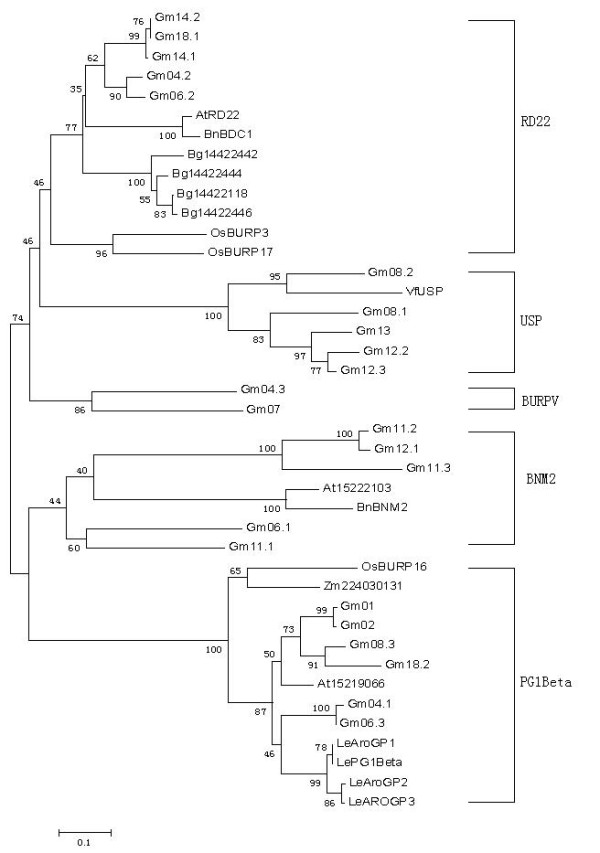
**Phylogenetic analysis of total 45 BURP domain-containing proteins from diverse plants**. The bootstrap values are indicated at each branch. The abbreviations of species names are as follows: At, *Arabidopsis thaliana*; Bn, *Brassica napus*; Bg, *Bruguiera gymnorrhiza*; Zm, *Zea mays*; Gm, *Glycine max*; Os, *Oryza sativa*; Vf, *Vicia faba*; Le, *Lycopersicon*.

**Figure 4:**
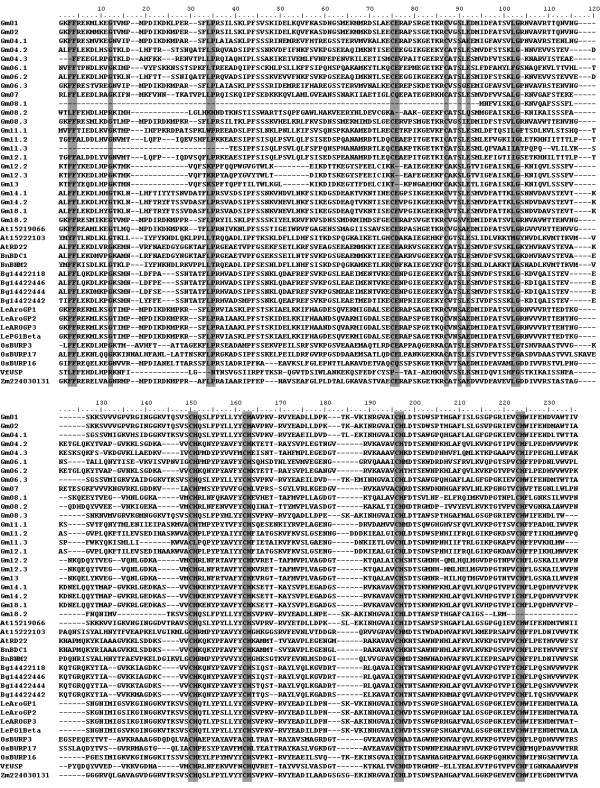
**Sequence alignment of BURP domain-containing proteins**. Conserved residues are shaded in grey.

Based on the protein sequences and the produced phylogenetic tree, 41 BURP proteins from different species (3 from *Arabidopsis thaliana*; 2 from *Brassica napus*; 4 from *Bruguiera gymnorrhiza*; 1 from *Zea mays*; 23 from *Glycine max*; 3 from *Oryza sativa*; 1 from *Vicia faba*; 4 from *Lycopersicon*.) were classified into 5 subfamilies, BNM2-like, USP-like, RD22-like, PG1β-like, and BURPV (Figure 3). The first four subfamilies had been defined before, but BURPV is new. Interestingly, all the BURPV members were from soybean. From the alignment of BURP domain sequences, several highly conserved residues were identified: two glycine residues, two F residues, two E residues, and four CH motifs (Figure 4). The conserved sequence of the BURP domain was described as X_5_-CH-X_10_-CH-X_23-27_-CH-X_23-26_-CH-X_8_-W, where X means any amino acid residue. However, not all sequences corresponded to this consensus. Gm08.1 with incomplete BURP domain lacked all the conserved residues but had all four CH motifs. In Gm14.1, Gm14.2 and Gm18.1 the last conserved residue W was replaced by F; and Gm18.2 lacked the last CH motif.

### Organ and tissue specific expression of *GmBURP *genes

**Figure 5:**
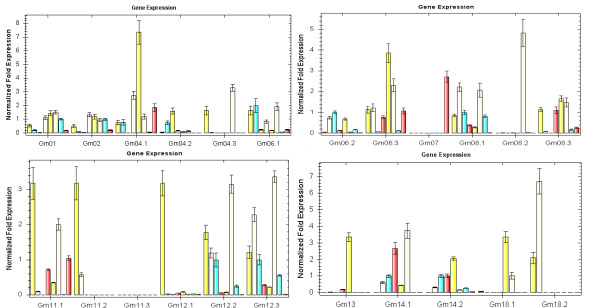
**Tissue-specific expression patterns of *GmBURP *genes**. The x-axis represents for different tissues or organs. The bars above each gene name indicate different tissues or organs. The order from left to right is: roots, stems, leaves, flowers, epicotyls, hypocotyls, cotyledons, and seeds. The y-axis shows the gene expression levels after normalization to reference gene *CYP2*.

Several genes of the BURP family have been noted for their differential expression patterns in various plant tissues and organs. As a way to reveal the expression pattern of each *GmBURP *gene, the transcript levels were determined in 8 different tissues and organs (root, stem, leaf, flower, epicotyl, hypocotyl, cotyledon, and seed) of soybean cultivar Zhonghuang13 using qRT-PCR analysis (Figure 5). The gene specific primers are listed in Table 2. The result showed that *GmBURP *genes vary widely in their specificities and in expression levels. According to their expression specificity *GmBURP *genes were divided into two groups. The first group (*Gm01*, *Gm02*, *Gm04.2*, *Gm04.3*, *Gm06.1*, *Gm06.2*, *Gm06.3*, *Gm08.1*, *Gm11.1*, *Gm12.1*, *Gm12.2*, *Gm12.3*, *Gm14.1*, *Gm14.2*, and *Gm18.1*) were expressed in all 8 tissues and organs but in different levels. Judging from their expression patterns, these genes may play roles in some basic metabolic pathways. *Gm04.3*, *Gm06.1*, *Gm11.1*, *Gm12.1*, *Gm12.2*, *Gm12.3*, and *Gm18.2 *were strongly expressed in roots. *Gm06.1*, *Gm08.1*, *Gm12.2*, *Gm12.3 *and *Gm18.2 *were strongly expressed in stems. *Gm04.2*, *Gm12.2 *and *Gm12.3 *were most strongly expressed in leaves. *Gm01*, *Gm02 *and *Gm14.1 *were strongly expressed in flowers, indicating that they may play a role in soybean sexual reproduction. *Gm01*, *Gm02*, *Gm04.3*, *Gm06.3 *and *Gm18.1 *were highly expressed in epicotyls. *Gm01*, *Gm04.3*, *Gm06.1*, *Gm06.3*, *Gm08.1*, *Gm11.1*, *Gm12.2*, *Gm12.3 *and *Gm14.1 *had high expression levels in hypocotyls. In seeds only two genes, *Gm04.3 *and *Gm06.3 *were highly expressed, indicating that they could have similar functions as *Gm07*.

**Table 2:** Primer sequences for real-time PCR analysis of BURP family genes in soybean

Gene name	Forward primers	Reverse primers
*Gm01*	GGGAATCAACGGTGGTAAAG	CGTGGCAGTAGTAAAGCAGATA
*Gm02*	ACGGGTCCAAGAAGAGTGTG	TGGCAGTAGTAGAGCAGGTAAGG
*Gm04.1*	GCGGAAGAAATGATAGGGTT	CGACACTGACTTGGTCACTTTT
*Gm04.2*	GAGTGAAGAAGTTATCAGGGGAC	ACCCCATTAGTACCCTCCAAA
*Gm04.3*	AGATGAAACGATCCTTGTTGCT	CTTTTCTTGAAGTGAGCATCCA
*Gm06.1*	CCTTACCCTTATGCAGTTTTTTAC	CTCTCCCTCCATTCTCACCTT
*Gm06.2*	TGCCACAAACAGAACTACCC	TCCAAAGGCACAGAGTAAGC
*Gm06.3*	GAGACTAACTCTGGATCGCAAA	GTTGAACTCCGACTGAAGAATG
*Gm07*	CTCAACAGAGAAAGAAAGGGAA	AAAGACAACATAAGGGTAACTCATT
*Gm08.1*	GGAACCAAAACTCAGGCACT	AAGGAAATGGCAAAGAGGG
*Gm08.2*	TTGGTAGCATCTGATGGAACTAA	CAAAATGACAAACAGGCACG
*Gm08.3*	TCCCTTACATGCTTTACTATTGTC	ACGCAGTGGTATCCAAGTGA
*Gm11.1*	CCCTTATACAGTTTTTTACTGCC	ACAACCATAGCATCCACCCT
*Gm11.2*	TGGGCTTCAGTTTCTTCCTC	TTGTACCATTCATCTTGTTTCTCA
*Gm11.3*	ACAACCTTATCCCTATGCGG	TCAGATGTGTCTAAATGGCAAAC
*Gm12.1*	TCATCCCCTACCATATCCCT	TCATCTCCATTCTCGCTACCTA
*Gm12.2*	CATGAAGTCCGTGAAACAACA	GCAAATAGCAAGTGCCTGAG
*Gm12.3*	GAGCAATACACTGTGGAAGGAG	TGTTTCACGGACTTTATGGC
*Gm13*	CAAGACAAAACCTAATGGAGCA	ACTTGAATGTTCTTTCCCAGC
*Gm14.1*	GAAGCAGGGTCAAAGCAGTT	GAACCTTAGGCACTTGAAACG
*Gm14.2*	CCTCATCTGACCCTTCCTTG	CAGTTGCTGATTCCATACCC
*Gm18.1*	TTGACTTTTCAACCTCCGTTT	CCTTTTACATTCTCTGTGGTCC
*Gm18.2*	CACAGAGGCTTTCAAGACAGG	GAAACCTCTTGGATGGGAAA

The second group of genes (*Gm04.1*, *Gm07*, *Gm08.2*, *Gm08.3*, *Gm11.2*, *Gm11.3*, *Gm13 *and *Gm18.2*) were not expressed in at least one of the eight selected tissues and organs. All the genes of this group, except for *Gm13*, were not expressed in leaves. *Gm11.3 *was not expressed in any of the eight tissues and organs. It may, however, be specifically expressed in certain tissues or development periods not studied here. *Gm07 *(*SCB1*), one of the well studied *GmBURP *genes from soybean, was highly expressed in seeds, very low expression in stems and cotyledons, and no expression in the other 5 tissues. *Gm08.2 *was highly expressed in hypocotyls, but not in leaves and roots, which suggested that it mainly functions at the early stages of soybean development. *Gm08.3 *had relatively high expression in epicotyls and flowers. *Gm13 *was highly expressed in epicotyls but not in seeds. As *Gm08.2 *it may mainly function at the early stages of plant development. *Gm11.2 *and *Gm18.2 *were expressed only in roots and stems. Since each member of this group lacked expression in one or more analysed tissues or organs, this group of genes may have more specific functions in soybean than the group 1 BURP genes.

All the *GmBURP *genes, except for *Gm18.2 *of the PG1β-like subfamily, were highly expressed in epicotyls. Only two BURPV subfamily members (*Gm04.3 *and *Gm07*) were highly expressed in seeds. Three genes, *Gm08.2*, *Gm12.2*, and *Gm12.3*, of the five *GmBURP *genes belonging to the USP-like subfamily were highly expressed in hypocotyls. Two BNM2-like subfamily genes *Gm06.1 *and *Gm11.1 *had high expression levels in hypocotyls, and *Gm06.1 *also had a high expression level in stems. Meanwhile, the expression levels of the RD22-like genes varied widely but all of them were expressed in all eight selected tissues and organs. More specifically, *Gm06.1 *was mainly expressed in stems, leaves and epicotyls; *Gm14.1 *was relatively high expressed in flowers and hypocotyls; *Gm14.2 *was mainly expressed in leaves, flowers, and hypocotyls; while *Gm18.1 *was highly expressed in epicotyls. The expression pattern indicates that members belonging to this subfamily may play significant roles in soybean, but it was postulated that they mainly function in different tissues or organs.

### Promoter *cis*-element identification and expression under stress treatments

Several genes of the BURP family have been reported to be stress related. Examples are *RD22 *from Arabidopsis which responds to drought, and *SALI3-2 *(*Gm12.3*) which is induced by aluminium.

**Figure 6:**
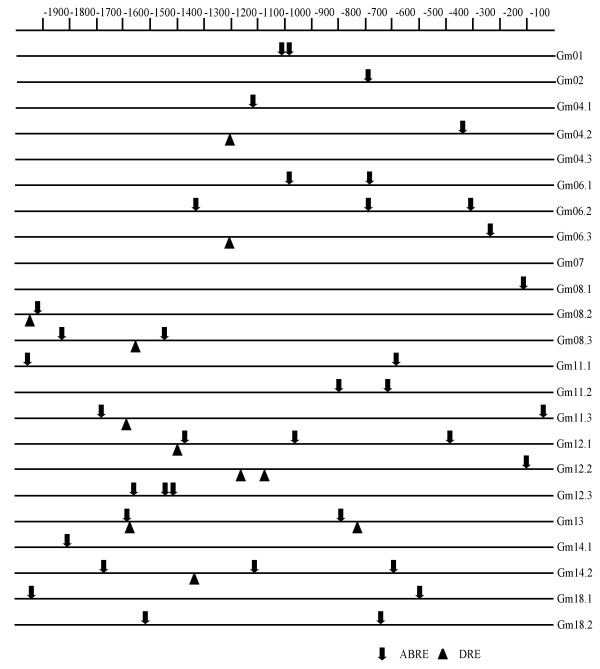
**Promoter sequence analysis of *GmBURP *genes**. The lines represent the promoter sequences. The scale bar on top is 100 bp. ABRE and DRE elements are indicated by **↓ **and ▲, respectively.

The online database PLACE was used to identify *cis*-elements for each *GmBURP *gene. 2000 bp upstream of the full-length cDNAs were searched against the database and two putative stress-responsive *cis*-elements ABRE (ABA responsive element) and DRE (dehydration-responsive element) [20,21] were found for most of the *GmBURP *genes (Figure 6). ABRE sequences were found in the promoter region of 21 of the 23 *GmBURP *genes except *Gm04.3 *and *Gm07*. DRE sequences were found in the promoter regions of 9 *GmBURP *genes (*Gm04.2*, *Gm06.3*, *Gm08.2*, *Gm08.3*, *Gm11.3*, *Gm12.1*, *Gm12.2*, *Gm13*, and *Gm14.2*). We noticed that ABRE elements were identified in the promoter regions of all members from BNM2-like, USP-like, and RD22-like subfamily but not BURPV subfamily. DRE sequences were identified in some members of all four subfamilies except BURPV. The results indicate that most of the *GmBURP *genes may be stress-relative.

**Figure 7:**
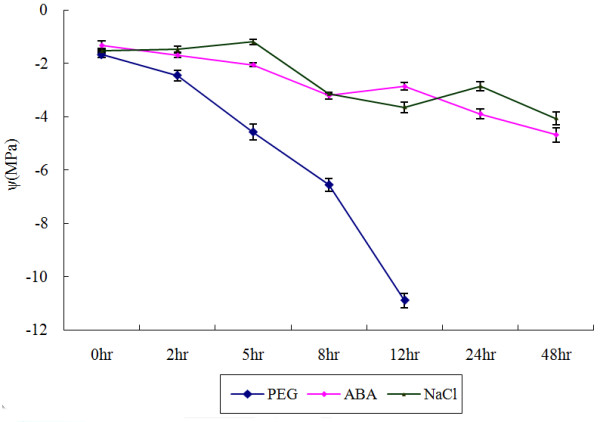
**Soybean leaf water potential (ψ) under three treatments: PEG, NaCl, and ABA**. The x-axis is the time courses of treatments. The y-axis is water potential of soybean leaf under stress treatments.

**Figure 8:**
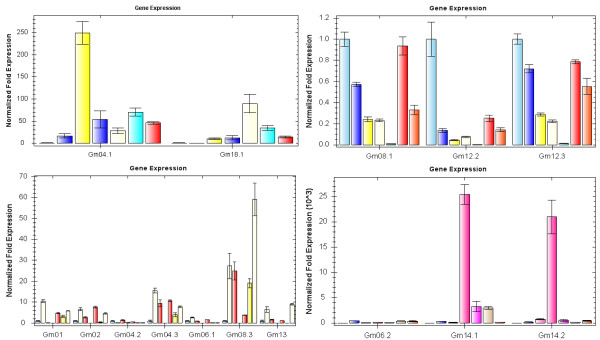
**Expression profiles of *GmBURP *genes under ABA treatment**. The x-axis is the time courses of ABA treatment. The bars from left to right indicate the time courses of treatment for 0 hr, 2 hr, 5 hr, 8 hr, 12 hr, 24 hr and 48 hr for each gene listed below x-axis. The y-axis is the expression levels after normalization to internal control gene *CYP2 *(data for genes not responding to stress treatments were omitted).

**Figure 9:**
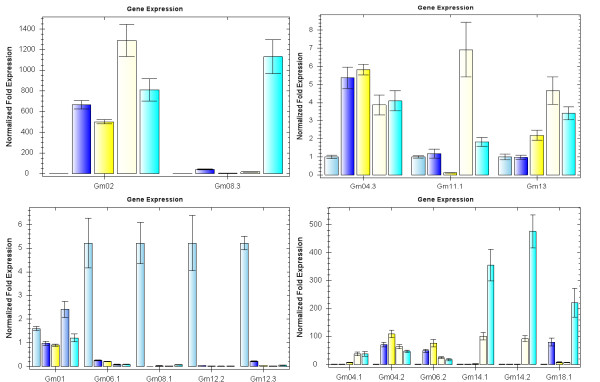
**Expression profiles of *GmBURP *genes under PEG treatment**. The x-axis is the time courses of drought treatment. The bars from left to right indicate the time courses of treatment for 0 hr, 2 hr, 5 hr, 8 hr, 12 hr for each gene listed below the x-axis. The y-axis is the expression levels after normalization to internal control gene *CYP2 *(data for genes not responding to drought treatment were omitted).

**Figure 10:**
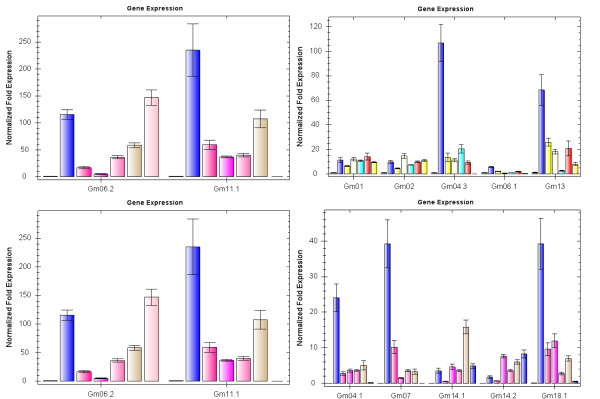
**Expression profile of *GmBURP *genes under NaCl treatment**. The x-axis is the time courses of NaCl treatment. The bars from left to right indicate the time courses of treatment for 0 hr, 2 hr, 5 hr, 8 hr, 12 hr, 24 hr and 48 hr for each gene listed below the x-axis. The y-axis is the expression levels after normalization to internal control gene *CYP2 *(data for genes not responding to NaCl treatment were omitted).

To support the predictions made by PLACE analysis, dehydration and salt-inducible *GmBURP *genes were screened. Water potential determination results showed that water potential of all samples was lowered under ABA, NaCl, or PEG treatments, indicating that plants were effectively stressed (Figure 7). qRT-PCR was employed to analyze the gene expression on transcriptional level under three different treatments (Figure 8, 9, 10). The result showed that 17 *GmBURP *genes responded to at least one of the stress treatments. For ABA treatment two genes, *Gm04.3*, and *Gm06.1*, and three genes, *Gm08.1*, *Gm12.2*, and *Gm12.3*, were up- and down-regulated, respectively, while 10 genes (*Gm01*, *Gm02*, *Gm04.1*, *Gm04.2*, *Gm06.2*, *Gm08.3*, *Gm13*, *Gm14.1*, *Gm14.2*, *Gm18.1*) were initially up-regulated and later down-regulated during the later stages of the treatment. After PEG treatment 5 genes (*Gm04.1*, *Gm08.3*, *Gm14.1*, *Gm14.2*, and *Gm18.1*) were up-regulated, and 4 genes (*Gm06.1*, *Gm08.1*, *Gm12.2 *and *Gm12.3*) were down-regulated, while 7 genes (*Gm01*, *Gm02*, *Gm04.2*, *Gm04.3*, *Gm06.2*, *Gm11.1*, and *Gm13*) were first up and then down-regulated. The expression patterns under NaCl were different: 3 genes, *Gm01*, *Gm02 *and *Gm14.2*, were up-regulated; 2 genes, *Gm12.1 *and *Gm12.2*, were down regulated; 9 genes (*Gm04.3*, *Gm04.1*, *Gm06.1*, *Gm06.2*, *Gm07*, *Gm11.1*, *Gm13*, *Gm14.1*, and *Gm18.1*) were first up and then down-regulated; 3 genes, *Gm04.2*, *Gm08.1*, and *Gm12.3*, were first down-regulated and then up-regulated.

Many *GmBURP *genes were regulated by more than one of the treatments. Among the 17 stress responsive *GmBURP *genes, 13 of them responded to all the three treatments; two of them (*Gm08.3 *and *Gm11.1*) respond to two different stresses; *Gm07 *and *Gm12.1 *only responded to NaCl treatment. More interestingly, two genes, *Gm04.1 *and *Gm07*, which were not expressed in leaves, were also stress responsive: *Gm04.1 *responded to all the three treatments while *Gm07 *responded to NaCl treatment only. We also noticed that all the members of BNM2-like (except for Gm11.3), RD22-like and BURPV were responsive to at least one treatment. More significantly, RD22-like subfamily genes responded to all three treatments. Some members of other subfamilies also responded to stress treatment: the PG1β-like subfamily had three genes (*Gm01*, *Gm02*, and *Gm08.3*) which were stress responsive, while the USP-like subfamily had four genes, *Gm12.2*, *Gm08.1*, *Gm12.3*, and *Gm13*, responded to at least one of the three treatments.

The results of qRT-PCR were not always consistent with these of the promoter region analysis. There were no ABRE or DRE elements detected in any of the promoter regions of BURPV family members, but the real-time PCR results showed that all the members of this subfamily were stress responsive. This strongly indicated that some unidentified stress responsive *cis*-elements may play an important role in regulating the soybean stress response.

## Discussion

### Structure characteristics of BURP proteins

BURP domain-containing proteins contain three or four distinct modules: (1) an N-terminal hydrophobic domain which is a presumptive signal peptide; (2) a short conserved segment; (3) an optional repetitive region which is unique to each member; and (4) the C-terminal BURP domain [1]. They were classified into four subfamilies by Granger [2], and the structure of the conserved BURP domain was described as CHX_10_CHX_25-27_CHX_25-26_CH [1]. The most obvious characteristics of the BURP domain are 2C residues and 4 CH motifs. More recent report showed that the distance between the last three CH motifs are not always 25-27 and 25-26, respectively, and the domain was newly described as X_5_-CH-X_10_-CH-X_23-37_-CH-X_23-26_-CH-X_8_-W [3]. However, the conserved residue F is replaced by W in three BURP domain-containing proteins, Gm14.1, Gm14.2, and Gm18.1. It was also noticed that all the three proteins were from RD22-like subfamily.

A lot of work has been done to reveal the structure of the BURP domain but still little is known about the function of each module. It is through the BURP domain, that SCB1 (Gm07) is localized on the cell wall [12]. The BURP domain of SALI3-2 (Gm12.3) was shown to be a key component for its tolerance to salt. Deletion of the signal peptide of SALI3-2 reduced salt tolerance of transgenic yeast [22]. So, it seems that these two parts are important for the function of BURP proteins. However, the mature PG1β contains only the repeated region after the cleavage site of its transit peptide, and the BURP domain. These two parts form a PG1 complex with catalytic PG2 polypeptide [4,23,24]. Despite the conservation of the BURP domains and their sequence similarities, the function of each BURP protein and the roles of each module seem to be greatly varied among plants.

### Evolution of BURP genes and divergence of their functions

In total 41 BURP domain-containing proteins from various plants were classified into 5 subfamilies. Members of RD22-like, BNM2-like, USP-like, and BURPV subfamilies were all from dicotyledons, while in PG1β subfamily members can come from both dicotyledon and monocotyledon plants.

The classification of the BURP protein genes may not be final, and it remains unknown how these subgroups evolved. By comparing the surrounding genomic sequences, Hattori [1] proposed that the exons coding the signal peptide and C-terminal BURP region may have been "shuffled" into the various modular protein structures. Hattori also suggested that in view of the highly repetitive nature of the BURP proteins, the amplification of short sequences has also been important during the evolution of the family. The PG1β subfamily BURP proteins from dicotyledons were clustered more closely than the two (Zm22403013 and OsBURP16) from monocotyledons which suggested that BURP genes existed before the divergence of the monocotyledon and dicotyledon lineages. Four GmBURPs (Gm01, Gm02, Gm08.3, and Gm18.2) are more closely clustered with each other than with At15219066 from *Arabidopsis *and two GmBURP proteins, Gm04.1 and Gm06.3, are more closely clustered than four proteins (LePG1β, LeAroGP1, LeAroGP2, and LeAroGP3) from tomato, indicating that duplications of some BURP genes in *Glycine max *happened earlier than the divergence of soybean and *Arabidopsis *or tomato. It is the same in BNM2-like, and USP-like subfamilies, suggesting that the divergence of soybean from faba bean (*Vicia faba L*.), and oilseed rape happened after the duplication of *GmBURP *genes.

The location of BURP genes in soybean may also give some insight into the evolution of the gene family. 19 out of 23 BURP genes were found located in duplicated regions (Table 1). 17 out of the 19 BURP genes (*Gm01*, *02*, *04.1*, *04.2*, *04.3*, *06.1*, *06.2*, *06.3*, *08.1*, *08.3*, *11.1*, *12.1*, *12.2*, *12.3*, *13*, *18.1*, and *18.2*) located in duplicate regions appear to originate from segmental duplications. *Gm11.2 *and *Gm11.3 *seem to originate from tandem duplications. Among the 19 BURP genes, *Gm04*.*1*, *Gm06*.*1*, *Gm08.1*, *Gm11*.*1 *and *Gm18*.*1 *were found as single copies, this is not surprising because mass gene losses and chromosome rearrangements following large-scale genome duplication have occurred in soybean [25], leading to losses of about 25% of duplicated genes [26]. The four genes, *Gm07*, *Gm08*.*2*, *Gm14*.*1 *and *Gm14*.*2*, which are located outside of the duplicated region, might have been produced by retrotransposition. The structure similarity and variation between genes located on the same chromosome and phylogenetic analysis might help to explain the order of duplication evens of the sister genes on the same chromosome. For example, *Gm04.2*, *Gm04.1*, and *Gm04.3 *located in different duplicate regions of the same chromosome, all have two introns flanked by three exons. However, phylogenetic analysis showed that *Gm04.2 *was more similar to *Gm06.2*, and *Gm04.1 *was close to *Gm06.3 *while *Gm04.3 *which is located relatively close to *Gm04.2 *had no duplicate genes on chromosome 6. It is possible that the duplication of the same ancestral gene on chromosome 4 created *Gm04.1 *and ancestor for *Gm04.3 *and *Gm04.2 *then they evolve independently. The intron and exon sequences for ancestor gene elongated for various reasons before it split into *Gm04.2 *and *Gm04.3*. Through fragmental duplication the two chromosome fragments, one contains *Gm04.1 *and the other contains *Gm04.2 *and *Gm04.3 *were independently copied to different part of chromosome 6. During the later evolution, the counterpart of *Gm04.3 *was lost and structures for the counterparts of *Gm04.1 *and *Gm04.2 *changed by deletion or insertion of other fragments or partial sequence repeats variations.

Genome blast did not identify any BURP genes in *Chlamydomonas reinhardtii *http://genome.jgi-psf.org/, while 18 BURP proteins from *Physcomitrella patens *subsp.*patens *were recorded in NCBI protein database. *Chlamydomonas reinhardtii *lives in lakes or other freshwaters, while the moss *Physcomitrella patens *grows on land, the differences in the BURP distribution indicate that BURP family genes appeared when plants start to move from water to land where the environment became more variable. The origin of BURP genes indicates this gene family may play a role in plant adaptation to adverse environments. According to the phylogenetic analysis, BURP genes from different species were classified into 5 subfamilies, this classification suggests that their functions diversified during evolution. *BNM2*, expressed during the start of microspore embryogenesis and the corresponding protein is confined to seeds [5,6,22], *VfUSP*, expressed during the early stages of zygotic embryogenesis [8] indicating that proteins in BNM2 and USP subfamilies may have similar functions. *PG1β*, the non-catalyticβ-subunit of the polygalacturonase isozyme (PG), and *SCB1 *from soybean seed coat suggest possibly similar functions for the other members in PG1β and BURPV subfamilies. RD22, from *Arabidopsis*, has been reported to be stress related. It is interesting that during embryogenesis, fruit ripening and seed development the cells need to lose water and store certain materials to fulfil the physical functions. During these processes an in-cell stress environment may be created. It seems possible that the diversified BURP members retain their original of stress response functions. For example, proteins belonging to RD22 subfamily mainly respond to stresses during the vegetative plant development, while members from USP or BNM2 subfamilies respond to stresses during plant reproduction.

### Organ or tissue specific expression of *GmBURP *genes and their expression under different stress treatments

In the current work, *GmBURP *gene expression pattern in different tissues and organs and under various stress conditions were analyzed by qRT-PCR. The result showed that some genes with high sequence similarity also have similar expression pattern in different tissues and organs or under different stress conditions. For instance, two genes *Gm01 *and *Gm02*, which are similar to each other, were expressed in all eight different tissues and organs and both had very low expression in leaves and seeds, while highly expressed in epicotyls and, under three different stress treatments they all had an up-regulated period. Two closely clustered genes, *Gm06.1*, and *Gm08.1*, of the BNM2-like subfamily, were highly expressed in stems and hypocotyls, and responded to all stress treatments. All the *GmBURP *genes from RD22-like subfamily showed no tissue specificity, and were responsive to all stress treatments. However, not all genes with similar sequences showed the same expression pattern. E.g. *Gm06.3 *and *Gm04.1 *are closely clustered and highly similar members of the PG1β-like subfamily. *Gm06.3 *was expressed in all eight tissues and organs and did not respond to any of the three stress treatments, while *Gm04.1 *was not expressed in leaves, and respond to all stress treatments. *Gm08.3 *and *Gm18.2 *from the same subfamily with high sequence similarity showed different expression patterns, too: *Gm08.3 *had no expression in leaves, but it responded to ABA or drought treatments. Although *Gm18.2 *did not expressed in leaves either, it was not responsive to any of the three treatments. Two genes, *Gm12.1 *and *Gm11.2*, from the BNM2-subfamily showed the same patterns as *Gm08.3 *and *Gm18.2*. Previously described examples also existed in the USP-like family. Sequence similarity indicates that these genes might be the result of relatively recent gene duplication events. The difference of tissue or organ specificity and stress response maybe result from different upstream or downstream regulatory elements or factors.

Through phylogenetic analysis we defined a new subfamily, BURPV, containing two members which are all from soybean. Promoter analysis revealed no ABRE or DRE elements was found in the upstream 2000 bp region of the full length cDNA. Interestingly, all the BURPVgenes responded to at least one stress treatment, suggesting the existence of some unknown stress related *cis*-element. One member of this new defined subfamily, SCB1 (Gm07), a seed coat specific protein has been reported to have very strict expression pattern [12], but we found that in addition to the strong expression in seeds, it was slightly expressed in cotyledons and stems. Interestingly, despite the lack of expression in leaves, SCB1 was up-regulated by NaCl stress. Another *GmBURP *gene, *Gm04.3*, from the new subfamily BURP may also play a role during seed development because it had a similar expression pattern to *SCB1 *which made it a candidate gene for study of the influence of stress treatments on seed development.

Soybean is very important for the society because it is a major source of oil and protein-rich food. However, the production of soybean is threatened by drought and restricted by poor soil quality. *RD22*, an *Arabidopsis *drought responsive gene has been reported for its stress response [15]. All the *GmBURP *genes belonging to RD22-like subfamily and most of *GmBURP *genes from other subfamilies are stress responsive. It's worth mentioning that the *GmBURP *genes *Gm04.1*, *Gm07*, and *Gm08.3*, which had no detectable transcription detected in unstressed leaves, were induced by stress treatments.

## Conclusions

The BURP domain-containing proteins are a large family of evolutionarily conserved proteins only found in plants. Members of the family had been reported to be involved in the reproductive development and stress resistance of plants. In this study of the complete soybean genome, we identified 23 BURP proteins. We also propose their classification based on the sequence alignment and previous reports which give insight into the evolutionary relationships of the genes from multiple plant species. In addition, qRT-PCR analysis revealed the expression patterns of these genes in different tissues and under different stress treatments. Most BURP genes showed no tissue specificity and respond to stress treatments. Clearly, there is a need to functionally validate the roles of those genes induced under different stress conditions. Another important finding of the work is that all the members in soybean that belong to RD22 subfamily showed strong response to all three stress treatments. This may indicate that this subfamily specifically acted as a defence against stress in soybeans, and it is likely to play similar roles in other plant species. The results described here will be helpful for the further study of the functions of BURP domain-containing proteins, and for the screening candidate drought resistance genes in soybean and in other plants.

## Methods

### Identification of BURP family genes in soybean

Four typical BURP gene sequences *BNM2 *[GenBank: AF049028], *USP *[Genbank: X13210], RD22 [GenBank: NM_122472], and *PG1β *[GenBank: M98466] were used to blast against the soybean genome database http://www.phytozome.net/soybean using TBLASTX program. For each query sequence several putative genes located on different chromosomes were found. A data file containing all the information of the target genes including location on the chromosomes, genomic sequences, full CDS sequences, and protein sequences was provided in the above website. Redundant genes were removed manually. The SMART database (http://smart.embl-heidelberg.de[27]) and the conserved domain database (CDD) http://www.ncbi.nlm.nih.gov/Structure/cdd/wrpsb.cgi were used to confirm each predicted BURP protein.

### Phylogenetic tree construction and sequence analysis

To investigate the molecular evolution and phylogenetic relationships among BURP proteins in plants, a phylogenetic tree was generated by multi-alignment of BURP domain sequences of all 23 BURP family proteins from soybean and 18 protein sequences from other plants (*Arabidopsis*, tomato, rice, faba, mangrove, bean, tomato, oilseed rape and maize). The SMART program was used to extract the protein sequences of the BURP domain for each protein [27], and then they were aligned using ClustalX1.83 [28]. The phylogenetic tree was constructed using software MEGA4.0 [29]. Online software Compute pI/Mw http://au.expasy.org/tools/pi_tool.html was used to predict the molecular weight and pI for each predicted BURP protein. SignalP 3.0 Server http://www.cbs.dtu.dk/services/SignalP/ and SMART were employed to predict possible signal peptides. Exon-intron organizations of *GmBURP *genes were determined by comparing predicted coding sequences (CDS) with their corresponding genomic sequences using software GSDS http://gsds.cbi.pku.edu.cn/. To analyze whether the motifs in each putative BURP gene implicated in stress responses, the online database PLACE (http://www.dna.affrc.go.jp/PLACE/index.html[30]) was employed by analyzing the 2000 bp upstream region of the predicted CDS.

### Plant growth and treatments

Seeds of soybean (*Glycine max L*.) cultivar Zhonghuang13 were germinated in pots containing soil collected from an experimental field of the Chinese Academy of Agricultural Sciences (Beijing, China), and the seedlings were grown in natural environment. To study tissue or organ specific expression, cotyledons, epicotyls, and hypocotyls were collected from five-day-old seedlings, while vegetative tissues such as leaves, stems, and roots were collected from 4-week-old seedlings. Flowers were collected when they were in full bloom. Seeds were collected 2 weeks after flowering. After samples were collected they were immediately frozen in liquid nitrogen and then stored at -80°C. For stress treatments, 4-week-old seedlings growing in a chamber were treated with different media containing 100 μm ABA, 150 mM NaCl, and 20% polyethylene glycol (PEG) 6000, respectively [31]. Seedlings without treatment were used as control. Leaves of the stress-treated plants were collected at time intervals of 0, 2, 5, 8, 12, 24, 48 h (PEG treated samples were collected within 0-12 hours because later collection they would dry up). After collecting all the samples were immediately frozen in liquid nitrogen and then stored at -80°C for RNA extraction. Water potential of soybean leaves was detected at each time interval for all the three treatments with WP4-T Dew Potentiameter (Decagon devices). Three replicates were detected for each sample.

### RNA extraction and synthesis of the first-strand cDNA

Total RNA was isolated from frozen samples by using TRIZOL Reagent (Invitrogen). Genomic DNA was removed by digesting each sample (10 μg of total RNA) with DNaseI (TIANGEN) according to the manufacture's instruction. After DNaseItreatment, 4 μl RNA of each sample was heated at 65°C for 7 min. Then the first-strand cDNA was synthesized in a 20 μl volume containing 0.5 μl AMV reverse transcriptase (Promega), 0.5 μl RNase inhibitor (Promega), 1 μl oligo dT primer, 2 μl dNTP mixture, 4 μl MgCl_2 _(25 mM), 2 μl 10×reverse transcriptase buffer and 4 μl heat treated RNA sample. Finally, the reaction mixture was incubated at 42°C for 50 min.

### Quantitative real-time polymerase chain reaction (qRT-PCR) analysis

Gene-specific primers were designed using Primer5.0 and their specificity was checked by observing the melting curve of the RT-PCR products. The Soybean constitutively expressed *CYP2 *(*cyclophilin*) gene was used as reference for normalization. The primers are as follows: sense: 5'-CGGGACCAGTGTGCTTCTTCA-3' and antisense: 5'-CCCCTCCACTACAAAGGCTCG-3' [32]. RT-PCR was performed in a 25 μl volume containing 12.5 μl 2×SYBR^® ^Premix Ex Taq™ (TaKaRa), 1 μl 50-fold diluted cDNA, 0.15 μl of each gene-specific primer and 11.2 μl ddH_2_O. The PCR conditions were as follows: 95°C for 3 min, 45 cycles of 15 s at 95°C, 57°C for 15 s and 72°C for 20 s. Three replicates were used for each sample. Reaction was conducted on a CFX96 Real-Time PCR Detection System (Bio-Rad). All data were analyzed using the CFX Manager Software (Bio-Rad).

## Authors' contributions

HX, YL, KW carried out all experiments and preparation of cDNA for qRT-PCR analysis. HX performed all qRT-PCR analysis and, in conjunction with YH, carried out and analyzed all the bioinformatics analysis. HX, YH, YY, and YG conceived the study, planned experiments, and helped draft the manuscript. All authors read and approved the final manuscript.
